# Supplementary material for: Staphylococcus aureus in the Processing Environment of Cured Meat Products
Source: Foods. 2023 May 26;12(11):2161. doi: 10.3390/foods12112161 (PMC10253079; doi:10.3390/foods12112161)
Supplement: Supplementary file 1 [file foods-12-02161-s001.zip › foods-2394182-supplementary.pdf]

Table S1. List of primers used in this study

	Gene	Primer name	Primer sequence 5′-3′	PCR product (bp)	Ta	Reference
S. aureus	16S	16S1	GTGCCAGCAGCCGCGGTAA	886		[60]
		16S2	AGACCCGGGAACGTATTCAC			
	*mec*A	mecA1	GGGATCATAGCGTCATTATTC	527		[60]
		mecA2	AACGATTGTGACACGATAGCC			
	nuc	nuc1	TCAGCAAATGCATCACAAACAG	255		[60]
		nuc2	CGTAAATGCACTTGCTTCAGG			
Enterotoxins	*sea*	sea3	CCTTTGGAAACGGTTAAAACG	127		[61]
		sea4	TCTGAACCTTCCCATCAAAAAC			
	seb	sebF	AAATACGTAGATGTGTTTGGAGC	374	58ºC	This study
		sebR	GGTGCAGGCATCATGTCATAC			
	sec	sec3	CTCAAGAACTAGACATAAAAGCTAGG	271		[61]
		sec4	TCAAAATCGGATTAACATTATCC			
	sed	sed3	CTAGTTTGGTAATATCTCCTTTAAACG	319		[61]
		sed4	TTAATGCTATATCTTATAGGGTAAACATC			
	see	seeF	TACCAATTAACTTGTGGATAGAC	171		[62]
		seeR	CTCTTTGCACCTTACCGC			
	seg	seg1	TGCTATCGACACACTACAACC	704		[63]
		seg2	CCAGATTCAAATGCAGAACC			
	seh	seh-1	GTCTATATGGAGGTACAACACT	213		[64]
		seh-2	GACCTTTACTTATTTCGCTGTC			
	sei	sei1	GACAACAAAACTGTCGAAACTG	630		[63]
		sei2	CCATATTCTTTGCCTTTACCAG			
	sej	seljF	GCATGATAAAAGAATCAGCCAGC	1338	58ºC	This study
		seljR	GCATATACGGGGGCGTTAC			
	*selk-selq**	QF	TAGCATATGCTGATGTAGGGGT	1200	58ºC	This study
		KR	CTTCTCCTTTTTTAGTGCCGTTATG			
	*ser***	serF	CCAGATCCAAGACCTGGAGAA	1123	58ºC	This study
		serR	CATCAGAACTGTTGTTCCGCT			

Antimicrobials	*bla*Z	blaZ1	ATGTAATTCAAACAGTTCACATGCC	701	57ºC	This study
		blaZ2	ATAGGTTCAGATTGGCCCTTAGG			
Biofilm	*ica*A	icaAF	CCTAACTAACGAAAGGTAG	1315 bp		[65]
		icaAR	AGATATAGCGATAAGTGC			
Virulence	*tst*	TST1	TTCACTATTTGTAAAAGTGTCAGACCCACT	180 bp		[66]
		TST2	TACTAATGAATTTTTTTATCGTAAGCCCTT			
	PVL	Pvl1	ATCATTAGGTAAAATGTCTGGACATGATCCA	433 bp		[46]
		Pvl2	GCATCAASTGTATTGGATAGCAAAAGC			
Enterotoxin gene cluster	*egc*	tSerF	AAAGTGTGTAGGCGCCAC	755 bp	56ºC	This study
		selOR	CATTATCCTTATACACAGCTACTCC			
		selOF	TGCAATATGTAGTGTAAACAATG	1380 bp	52ºC	This study
		selmvR3	CATTAACCCAAAGATTAATAGGAATA			
		selmvF	GCTGAATTTAAGAACGTTGA	1064 bp	50ºC	This study
		seiR	TGTTATTATGACCATATATATTGTATTC			
		seiF1	GCCACTTTATCAGGACAATAC	1736 bp	51ºC	This study
		selnR2	GTTAAGCCTACACATTTATTTCC			
		2427F†	AGGACAATACTTAAATTCTGCT	1338 bp	53ºC	This study
		3758R†	TCTCCCTCAATTTTTTGGT			
		3082F†	GAATTCACTGGTCTAATGGAT	1153 bp	53ºC	This study
		4215R†	TCTCTTCATCTAATTGATTTCCA			
		selnF	CAGATTTAGCAAATCAATTTAAAG	1311 bp	55ºC	This study
		segR3	GTCTTGCTTTGTAATCTAGTTC			
		segF	CTCCACCTGTTGAAGGAAGAG			
		HPR1***	TATCATTTCCACTGGAGAATCATG	1338 bp	56ºC	This study
		HPR2***	GTGATATCAAACCAAACAGACC	870 bp	56ºC	This study

*Amplifies 1200 bp comprising part of both genes (*selk* and *selq*).

** *ser* gene is located along with *sej*, these primers amplify a fragment of both genes.

† Amplifies a fragment of *egc* cluster reluctant in several strains with primers seiF-selnR2.

*** HPR1 or HPR2 were used as reverse primers with segF depending on the strain comprising part of the mentioned genes.
